# Thylakoid redox signals are integrated into organellar-gene-expression-dependent retrograde signaling in the *prors1-1* mutant

**DOI:** 10.3389/fpls.2012.00282

**Published:** 2012-12-27

**Authors:** Luca Tadini, Isidora Romani, Mathias Pribil, Peter Jahns, Dario Leister, Paolo Pesaresi

**Affiliations:** ^1^Plant Molecular Biology (Botany), Department Biology I, Ludwig-Maximilians-Universität MünchenMunich, Germany; ^2^Plant Biochemistry, Heinrich-Heine-University DüsseldorfDüsseldorf, Germany; ^3^Dipartimento di Bioscienze, Università degli studi di MilanoMilan, Italy

**Keywords:** gene expression, organelle, translation, light, redox, retrograde signaling

## Abstract

Perturbations in organellar gene expression (OGE) and the thylakoid redox state (TRS) activate retrograde signaling pathways that adaptively modify nuclear gene expression (NGE), according to developmental and metabolic needs. The *prors1-1* mutation in Arabidopsis down-regulates the expression of the nuclear gene *Prolyl-tRNA Synthetase1* (*PRORS1*) which acts in both plastids and mitochondria, thereby impairing protein synthesis in both organelles and triggering OGE-dependent retrograde signaling. Because the mutation also affects thylakoid electron transport, TRS-dependent signals may likewise have an impact on the changes in NGE observed in this genotype. In this study, we have investigated whether signals related to TRS are actually integrated into the OGE-dependent retrograde signaling pathway. To this end, the *chaos* mutation (for chlorophyll a/b binding protein harvesting-organelle specific), which shows a partial loss of PSII antennae proteins and thus a reduction in PSII light absorption capability, was introduced into the *prors1-1* mutant background. The resulting double mutant displayed a *prors1-1*-like reduction in plastid translation rate and a *chaos*-like decrease in PSII antenna size, whereas the hyper-reduction of the thylakoid electron transport chain, caused by the *prors1-1* mutation, was alleviated, as determined by monitoring chlorophyll (Chl) fluorescence and thylakoid phosphorylation. Interestingly, a substantial fraction of the nucleus-encoded photosynthesis genes down-regulated in the *prors1-1* mutant are expressed at nearly wild-type rates in *prors1-1 chaos* leaves, and this recovery is reflected in the steady-state levels of their protein products in the chloroplast. We therefore conclude that signals related to photosynthetic electron transport and TRS, and indirectly to carbohydrate metabolism and energy balance, are indeed fed into the OGE-dependent retrograde pathway to modulate NGE and adjust the abundance of chloroplast proteins.

## Introduction

Several features link mitochondria and plastids within the plant cell. Both organelles maintain and express genetic information, conduct electron transport functions, and are metabolically interdependent (Woodson and Chory, 2008).

In addition, the majority of mitochondrial and plastid proteins are nucleus-encoded (Kleine et al., 2009a). Therefore, to ensure that the multiprotein complexes essential for photosynthesis and respiration are assembled correctly, the activities of both organelle types must be closely coordinated with that of the nuclear genome. Signals from the organelles to the nucleus are collectively referred as retrograde signals and can largely be grouped into two categories. (1) Biogenic control, i.e., developmental control of organelle biogenesis needs to be appropriately staged and the required subunits and cofactors need to be present in correct stoichiometry for accurate assembly; and (2) operational control, representing rapid adjustments that are made to energy metabolism in response to environmental and developmental constraints to maintain optimal production and both limit and repair damage induced by oxidative stress (reviewed in Leister, 2005; Pesaresi et al., 2007; Pogson et al., 2008; Woodson and Chory, 2008; Barajas-Lopez et al., in press). Given the complexity of organellar functions, a variety of interlinked retrograde pathways can be expected (Kleine et al., 2009b; Leister, 2012).

Several metabolites have been proposed to act as messenger molecules during retrograde signaling, including (1) tetrapyrroles (Mg-protoporphyrin IX or heme) (Strand et al., 2003; Woodson et al., 2011); (2) phosphonucleotide 3′-phosphoadenosine 5′-phosphate (PAP) (Estavillo et al., 2011); (3) β-cyclocitral (Ramel et al., 2012); and (4) methylerythritol cyclodiphosphate (MEcPP) (Xiao et al., 2012). Moreover, plastid retrograde signaling has also been associated with responses to perturbations in photosynthetic electron transport [changes in thylakoid and stromal redox state, accumulation of the reactive oxygen species (ROS) ^1^O_2_ and H_2_O_2_ (reviewed in Apel and Hirt, 2004; Oelze et al., 2008)], as well as in organellar gene expression (OGE), involving the pentatricopetide protein GUN1, abscisic acid (ABA) and the transcription factors PTM and ABI4 (Sullivan and Gray, 1999; Koussevitzky et al., 2007; Sun et al., 2011).

Recently, the role of Mg-protoporphyrin IX (Mg-ProtoIX) as a plastid signal has been questioned, since its accumulation following norflurazon treatment could not be correlated with changes in NGE (Mochizuki et al., 2008; Moulin et al., 2008). Consequently, it was suggested that either rapid changes in the flux through the tetrapyrrole pathway, or the accumulation of Mg-ProtoIX in a specific cellular compartment could be the origin of the plastid signal (Mochizuki et al., 2008; Moulin et al., 2008); however, these aspects deserve further investigations. Similarly, the role of ROS as classical retrograde signals is debated, because they are either probably too short-lived to reach the nucleus, as in the case of singlet oxygen ^1^O_2_, or too unspecific (H_2_O_2_) to act as information carriers (Moller and Sweetlove, 2010).

Beside the uncertainty on messenger molecules, little is also known about the extent to which different signals can be integrated into common pathways. The first insights into signaling pathways that serve to integrate chloroplast and mitochondrial activities with NGE were obtained through the characterization of mutant alleles of *PRORS1* (*At5g52520*), an Arabidopsis gene coding for a prolyl-tRNA synthetase that is imported into both chloroplasts and mitochondria (Pesaresi et al., 2006). The leaky *prors1-1* mutant allele exhibited defects in photosynthesis due to the simultaneous impairment of translation in plastids and mitochondria. Concomitantly, a specific and marked drop in the levels of transcripts of nuclear genes for proteins involved in the light reactions of photosynthesis was observed, implying that the activity of the OGE-dependent retrograde signaling pathway was altered. To investigate the specific roles of protein synthesis in mitochondria and chloroplasts in regulating nuclear photosynthetic gene expression, Arabidopsis mutants altered in mRNA translation in either mitochondria (*mrpl11-1*) or plastids (*prpl11-1)* were isolated (Pesaresi et al., 2001, 2006). Comparison of the transcript profiles of *prors1-1*, *mrpl11-1*, and *prpl11-1* mutants and the double mutant *mrpl11-1 prpl11-1* showed that plastids and mitochondria generate signals which act synergistically to modulate nuclear photosynthetic gene expression.

In this study, we have investigated the extent to which signals related to photosynthetic electron transport contribute to the OGE-dependent retrograde signaling pathway, by introducing the *chaos* mutation (Klimyuk et al., 1999) into the *prors1-1* mutant background. In the *chaos* mutant, the *CAO* gene (*At2g47450*), which codes for the chloroplast recognition particle cpSRP43, is inactivated. CpSRP43 together with cpSRP54 form the chloroplast signal recognition particle complex (Keegstra and Cline, 1999), required for the integration of the PSII antenna proteins (Lhcb proteins) into the thylakoid membranes (Schuenemann et al., 1998; Amin et al., 1999; Klimyuk et al., 1999). Consequently, *chaos* plants are characterized by reduced PSII antenna size, as manifested by decreased Chl *b* and Lhcb protein contents, together with reduced levels of oxygen production and growth rate (Amin et al., 1999; Klimyuk et al., 1999). Moreover, the reduced light absorption is also associated with significantly lower foliar H_2_O_2_ levels than in wild type (WT), and is responsible for less photobleaching of leaves, lower induction of cytosolic ascorbate peroxidases, and lower degree of photoinhibition, indicating that *chaos* chloroplasts are maintained in a more oxidized state than WT (Klenell et al., 2005).

The *prors1-1 chaos* double mutant was compared with each single mutant in relation to rates of translation in plastids, photosynthetic performance, and NGE. The results obtained imply that signals related to photosynthetic electron transport, and indirectly to carbon metabolism and energy balance, can indeed be integrated into the OGE-dependent retrograde pathway.

## Materials and methods

### Plant material, propagation, and growth measurements

The *prors1-1* mutant allele and its detection by PCR are described in Pesaresi et al. (2006). In particular, the mutation is caused by a T-DNA insertion (*pAC106*) at −44 bp from the translation starting codon. The gene-specific primers prors1-sense (5′-AACCAAGCATGAGTTTCTCG-3′) and prors1-antisense (5′-ATCCGGAAAGAGGTCTGTTC-3′) were employed to detect the WT *PRORS1* allele; the T-DNA-specific primer T9697 (5′-CTCTTTCTTTTTCTCCATATTGACCAT-3′) and prors1-antisense were used to identify the *prors1-1* mutant allele. The *chaos* mutant used in this study was identified in a population mutagenized with the *En* transposon (Wisman et al., 1998) based on its photosynthetic performance and leaf pigment composition (Varotto et al., 2000). The mutant allele carries an *En* insertion (which is stable because of 249-bp deletion at the left border) at position +149 (relative to the start codon) in the single-exon gene *CAO*. The gene-specific primers cao-sense (5′-ATGCAAAAGGTCTTCTTGGC-3′) and cao-antisense (5′-CCTCTCTCGTCTTCCACTTC-3′) were employed in PCRs to detect the WT *CAO* allele; the *En*-specific primer EnR (5′-GAGCGTCGGTCCCCACACTTCTATAC-3′) and cao-antisense were used to identify the *chaos* mutant allele. The *prors1-1 chaos* double mutant was obtained by crossing *prors1-1* and *chaos* single mutants and PCR-genotyping F2 individuals. *Arabidopsis thaliana* Heynh. WT (Col-0) and mutant plants were grown under controlled growth chamber conditions as described (Pesaresi et al., 2009). The method used for growth measurement has been described before (Leister et al., 1999).

### Nucleic acid analysis

*A. thaliana* DNA was isolated as described (Ihnatowicz et al., 2004). For RNA analysis, total leaf RNA was extracted from fresh tissue using the TRIzol reagent (Invitrogen, Germany). Northern analysis was performed under stringent conditions, according to Sambrook and Russell (2001). Probes complementary to nuclear or chloroplast genes were used for the hybridization experiments. Primers used to amplify the probes are listed in Table 1. All probes used were cDNA fragments labeled with ^32^P. Signals were quantified with a phosphoimager (Typhoon; GE Healthcare, Munich, Germany) using the program ImageQuant (version 1.2; Molecular Dynamics). For quantitative real-time PCR (qRT-PCR) analysis, 4 μg aliquots of total RNA, treated with DNase I (Roche Applied Science) for at least 30 min, were utilized for first-strand cDNA synthesis using iScript reverse transcriptase (Bio-Rad) according to the supplier's instructions. The qRT-PCR profiling was carried out on an iCycler iQ5 real-time PCR system (Bio-Rad), using the oligonucleotide sequences reported in Table 1. Actin was used as internal standard. Data from three biological and three technical replicates were analyzed with Bio-Rad iQ5 software (version 2.0).

**Table 1:** **Oligonucleotide sequences employed for gene expression analysis**.

**Gene**	**Sense primer**	**Antisense primer**
*psaB (ATCG00340)*	GTATTGCTACCGCACATGAC	CCACGAAACTCTTGGTTTCC
*psbA (ATCG00020)*	CGGCCAAAATAACCGTGAGC	TATACAACGGCGGTCCTTATG
*RbcL (ATCG00490)*	CGTTGGAGAGACCGTTTCTT	CAAAGCCCAAAGTTGACTCC
*RbcS (AT1G67090)*	ATGGCTTCCTCTATGTTCTC	CGGTGCATCCGAACAATGGA
*Lhca1(AT3G54890)*	GTCAAGCCACTTACTTGGGA	GGGATAACAATATCGCCAATG
*Lhca2(AT3G61470)*	GAGTTCCTAACGAAGATCGG	AAGATTGTGGCGTGACCAGG
*Lhca3(AT1G61520)*	AGGCTGGTCTGATTCCAGCA	ACTTGAGGCTGGTCAAGACG
*Lhca4(AT3G47470)*	TGAGTGGTACGATGCTGGGA	GTGTTGTGCCATGGGTCAGA
*Lhcb1(AT1G29910)*	GACTTTCAGCTGATCCCGAG	CGGTCCCTTACCAGTGACAA
Lhcb1(AT1G29910)^*^	AGAGTCGCAGGAAATGGG	AAGCCTCTGGGTCGGTAG
*Lhcb2(AT2G05070)*	GAGACATTCGCTAAGAACCG	CCAGTAACAATGGCTTGGAC
Lhcb2(AT2G05070)^*^	GCTATCCAACAATCCTCCTTC	CCAGTTAAGTAAGACGGTGTG
*Lhcb3(AT5G54270)*	GGAGATGGGCAATGTTGGGA	TAGTTGCGAAAGCCCACGCA
Lhcb3(AT5G54270)^*^	CCGTGTGGACTTCAAAGAACC	CGCCAACACCATCAAGACC
*Lhcb4(AT3G08940)*	AGCTAGTGGATGGATCATCT	CAGGAGGAAGAGAAGGTATC
*PsaD1(AT4G02770)*	AAGCCGCCGGGATCTTCAAC	CTAAGCCTTGTCCCCAAAGC
*PsaE1(AT4G28750)*	ATGGCGATGACGACAGCATC	TGTTGGTCGATATGTTGGCG
*PsaF (AT1G31330)*	GTTCGACAACTACGGGAAGT	CTTAGCAATGAGATCACCAT
*PsaK (AT1G30380)*	ATGGTCTTCG AGCCACCAAA	CGTTCAGGTGCATGAGAATA
*PsaO (AT1G08380)*	ATGGCAGCAACATTTGCAAC	GTAATCTTCAGTCCTGCCCT
*PsbO2 (AT3G50820)*	AGACGGAAGCGTGAAGTTCA	CAATCTGACCGTACCAAACC
*PsbT2 (AT3G21055)*	ATGGCGTCAATGACCATGAC	CAGTTACGGCATATCTTGGC
*PsbX (AT2G06520)*	ATGGCTTCTACCTCCGCGAT	TAGGTTCTCTTGACAGGGTC
*Ferritin1(AT5G01600)*	ATGGCCTCAAACGCACTCTC	ATGCCCTCTCTCTTCCTCAC
Ferritin1(AT5G01600)^*^	TAAACGTTCACAAAGTGGCC	TAGAGGTCCAAGTCTAGTTC
*AOX1(AT1G32350)*	GGTTCTGAATGGAAGTGGAAC	GGAGCTGGAGCTTCCTTTAGT
AOX1(AT1G32350)^*^	CCGCACTCTTCGACCGGTAC	GCTGAACCGTCCGGTTTAGT
*2CPA (AT3G11630)*	ATGGCGTCTGTTGCTTCTTC	TGCAAGGTGAGAGAACACAC
2CPA (AT3G11630)^*^	CCGGATTTGCTCGACGCTCT	CAACTTCTCAAATTCTGAATGC
*CAT1(AT1G20630)*	CTTCTTTGACTGTCGGAACTC	CCAGTATCCTCCAGTTCTCC
CAT1(AT1G20630)^*^	ACAACAGTGCAGACACACGC	AGCGCTTGAAGGACGAACCC
ACTIN^*^	ACTACTGGTATTGTGTTGGACTC	CCCTTACGATTTCACGCTCTG

**Asterisk:** indicates primer pairs employed for qRT-PCR analyses.

### PAGE and immunoblot analyses

Leaves were harvested from plants at the 6-leaf rosette stage, and thylakoids were prepared as described (Bassi et al., 1985). For SDS-PAGE, thylakoid proteins isolated from equal amounts of leaf material (fresh weight) were fractionated on denaturing Tris-glycine SDS-PAGE gels (with 12% PA) and the protein content was stained with colloidal Coomassie blue (G 250).

For immunoblot analyses, total proteins were prepared from plants at the 6-leaf rosette stage (Martinez-Garcia et al., 1999), then fractionated by SDS-PAGE (on 12% polyacrylamide gels) (Schägger and von Jagow, 1987). Subsequently, proteins were transferred to poly(vinylidene difluoride) membranes (Ihnatowicz et al., 2004), and replicate filters were probed with appropriate antibodies. Signals were detected by enhanced chemiluminescence (GE Healthcare). Thylakoid protein phosphorylation was monitored with a phosphothreonine-specific antibody (Cell Signaling Technology) in total leaf protein extracts obtained from WT and mutant plants kept overnight in the dark and then exposed to light (80 μmol photons m^−2^ s^−1^) for 4 h.

Coomassie-stained gels and immunoblots were scanned and quantified using ImageQuant (version 1.2; Molecular Dynamics).

### *In vivo* translation assay

The *in vivo* translation assay was performed essentially as in Pesaresi (2011). Twelve leaf discs (4 cm in diameter) were incubated in a buffer containing 20 μg/ml cycloheximide, 1 mM K_2_HPO_4_–KH_2_PO_4_ (pH 6.3), and 0.1% (w/v) Tween-20 to block cytosolic translation. [^35^S]methionine was added to the buffer (0.1 mCi/ml) and infiltrated into the discs under vacuum. Leaves were exposed to light (20 μmol photons m^−2^ s^−1^) and four leaf discs were collected at each time point (5, 15, and 30 min). Total proteins were extracted as described above and fractionated by Tris-glycine SDS-PAGE (12% PA). Signals were detected and quantified using the phosphoimager and the ImageQuant program as described above.

### Chl fluorescence, oxygen evolution, and pigment analyses

*In vivo* Chl a fluorescence of leaves was measured using the Dual-PAM-100 (Walz, Effeltrich, Germany) as described (Pesaresi et al., 2009). Five plants of each genotype were analyzed and average values plus standard deviations were calculated. Plants were first dark-adapted for 30 min and minimal fluorescence (*F*_0_) was measured. Then pulses (0.8 s) of saturating white light (5000 μmol photons m^−2^ s^−1^) were used to determine the maximum fluorescence (*F*_*M*_), and the ratio (*F*_*M*_ − *F*_0_)/*F*_*M*_ = *F*_*V*_/*F*_*M*_ (maximum quantum yield of PSII) was calculated. An 8-min exposure to actinic red light (37 μmol photons m^−2^ s^−1^) served to drive electron transport between PSII and PSI at steady state. In particular, the employed routine allowed to measure the steady-state fluorescence (*F*_*S*_) and the maximum fluorescence after light adaptation (*F*_*M*′_) (saturation pulse, 0.8 s, 5000 μmol photons m^−2^ s^−1^) every 20 s. The ratio (*F*_*M*′_ − *F*_*S*_)/*F*_*M*′_ gives the effective quantum yield of PSII (Φ_II_),while the excitation pressure parameter 1-qL reflects the size of the reduced fraction of Q_A_. The coefficient of photochemical quenching, qL, was calculated as (*F*_*M*′_ − *F*_*S*_)/(*F*_*M*′_ − *F*_0′_) × *F*_0′_/*F*_*S*_ (Kramer et al., 2004), with *F*_0′_ being the minimum fluorescence after removal of the illumination. A 2-min dark period was also employed to monitor the recovery to the maximum quantum yield of PSII.

*In vivo* Chl a fluorescence of whole plants was recorded using an imaging Chl fluorometer (Imaging PAM; Walz, Germany). Dark-adapted plants were exposed to a pulsed, blue measuring beam (1 Hz, intensity 4; *F*_0_) and a saturating light flash (intensity 4) to obtain *F*_*V*_/*F*_*M*_. A 10-min exposure to actinic light (80 μmol photons m^−2^ s^−1^) was then used to calculate Φ_II_ at the steady state.

Rates of oxygen evolution from leaf discs were measured with a Clark-type oxygen electrode (Hansatech Instruments Ltd.) as described by Havaux and Devaud (1994). In particular, measurements were performed at saturating CO_2_ concentration by employing a bicarbonate/carbonate buffer. The light limited rates of photosynthetic O_2_ evolution were measured at 80 μmol photons m^−2^ s^−1^. Pigments were analyzed by reversed-phase HPLC (Farber et al., 1997).

## Results

### Light absorption and oxygen evolution are reduced in *prors1-1 chaos* leaves

**Figure 1:**
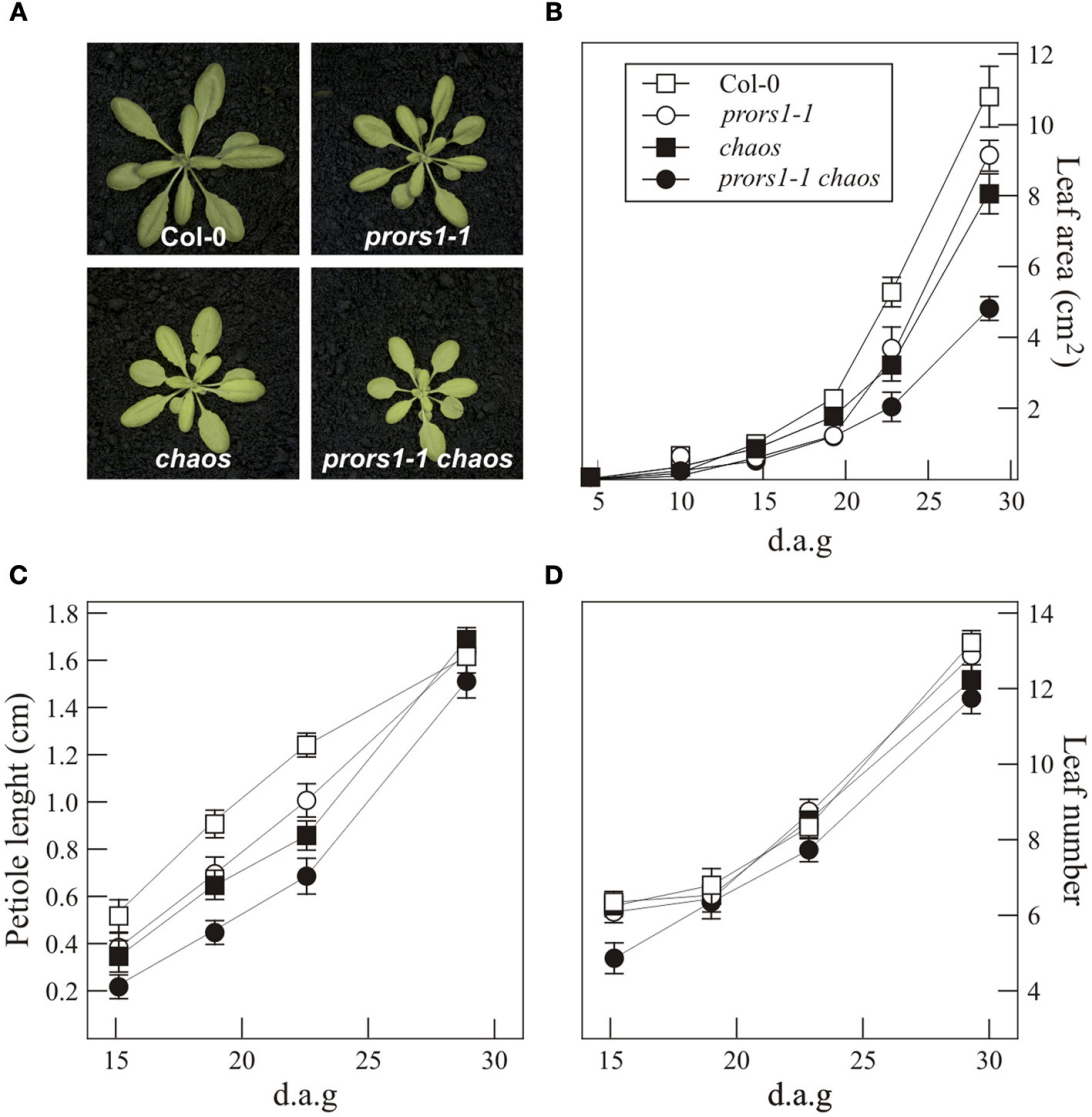
**Phenotypes of mutant (*prors1-1*, *chaos*, and *prors1-1 chaos*) and WT (Col-0) plants. (A)** The different genotypes were grown for 4 weeks in the growth chamber. **(B)** The growth rates of the different genotypes were measured from 4 d to 28 d after germination (d.a.g.). **(C)** Petiole length was measured by monitoring the petiole of the fifth true leaf from 15 to 28 d.a.g. **(D)** The number of true leaves was determined during the period from 15 to 28 d.a.g. Each point reported in **(B)**, **(C)**, and **(D)** is based on the determination of average leaf area, petiole length and leaf number, respectively, in at least 12 individuals. Bars indicate standard deviations.

The double mutant *prors1-1 chaos* was generated as described in “Materials and Methods.” Like the corresponding single mutants *prors1-1* and *chaos*, the *prors1-1 chaos* double mutant has pale-green cotyledons and leaves, and is smaller than WT plants of the same age (Figure 1A). Quantification of growth rates by non-invasive image analysis under growth chamber conditions showed that while *prors1-1* and *chaos* mutants were about 20 and 30% smaller, respectively, than WT plants at 4 weeks of age, the *prors1-1 chaos* double mutant displayed a size reduction of more than 50% relative to WT plants (Figures 1A,B). In addition, reductions in petiole length could be observed in *prors1-1*, *chaos* and, more markedly, in *prors1-1 chaos* mutants during the first stages of plant development, whereas such differences were less pronounced in adult plants (Figure 1C). Col-0, *prors1-1*, and *chaos* plants showed also a very similar number of leaves throughout the life cycle, whereas the leaf number was slightly decreased in *prors1-1 chaos* plants, particularly during the first 2 weeks after germination (Figure 1D).

**Figure 2:**
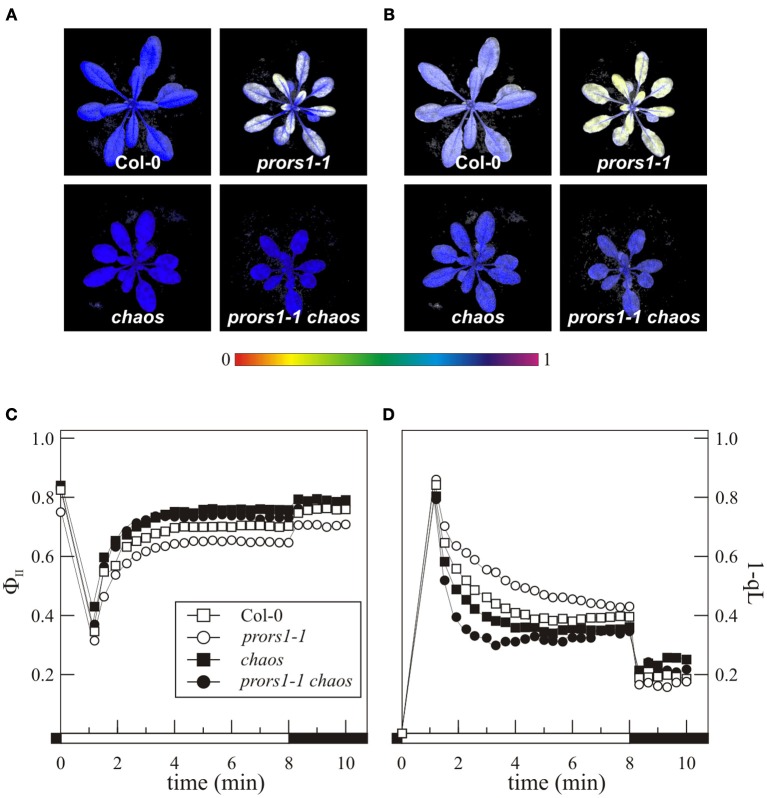
**Photosynthetic performance of mutant (*prors1-1*, *chaos*, and *prors1-1 chaos*) and WT (Col-0) plants.** The photosynthetic parameters *F*_*V*_/*F*_*M*_ **(A)** and Φ_II_ **(B)** were measured in the different genotypes, by employing an imaging Chl fluorometer, as described in “Materials and Methods.” Signal intensities for *F*_*V*_/*F*_*M*_ and Φ_II_ are indicated according to the color scale at the bottom of the panel. *F*_*V*_/*F*_*M*_ (time 0), Φ_II_ **(C)** and 1-qL **(D)** were also measured using the Dual-PAM-100, as described in “Materials and Methods.” Black bars indicate dark periods, and white bars light periods. Standard deviations were always below 10%.

Four-week-old WT and mutant plants were also subjected to Chl a fluorescence measurements to monitor photosynthetic performance (Figure 2). The data showed a lower maximum quantum yield of PSII (*F*_*V*_/*F*_*M*_) in *prors1-1*, whereas values higher than in WT were observed in *chaos* leaves. Accordingly, the *chaos* mutation was able to restore *F*_*V*_/*F*_*M*_ to the WT level in *prors1-1 chaos* leaves (Figures 2A,C). When the Φ_II_ parameter, reflecting the effective quantum yield of PSII, was taken into account, it could be also observed that *prors1-1* plants showed reduced Φ_II_ values with respect to Col-0, whereas *chaos* and *prors1-1 chaos* values were even higher than those of WT leaves (Figures 2B,C). In agreement with these observations, a decrease in the degree of reduction of Q_A_ (the primary electron acceptor of PSII) and the plastoquinone (PQ) pool (measured as 1-qL; a parameter frequently used for an indirect estimation of the redox state of the PQ pool; Kramer et al., 2004; Jung et al., 2010) was observed in *chaos* and *prors1-1 chaos* leaves (Figure 2D), suggesting a reduced net electron injection into the thylakoid transport chain, caused by *chaos* mutation. This aspect was investigated further by measuring oxygen production with a Clark-type oxygen electrode under standard lighting conditions. The levels of oxygen production per unit of leaf area were always found to be lower in *chaos* and *prors1-1 chaos* leaves than in Col-0 or the *prors1-1* single mutant (*chaos*, 0.014 ± 0.002 μmol m^−2^ h^−1^; *prors1-1 chaos*, 0.011 ± 0.003; *prors1-1*, 0.023 ± 0.004; Col-0, 0.029 ± 0.003).

To quantify the alteration in leaf coloration in *prors1-1*, *chaos*, and *prors1-1 chaos* plants, leaf pigments were analyzed by HPLC (Table 2). As expected, the total Chl content (Chl a+b) was reduced by about 57 and 66% in *chaos* and *prors1-1 chaos* leaves, respectively, and the Chl a/b ratio was 3.69 in *chaos* and 3.01 in *prors1-1 chaos* leaves, with respect to 2.71 in Col-0. In addition, carotenoids that bind specifically to PSII antenna proteins (Lhcb), such as neoxanthin (Nx), lutein (Lut), and the VAZ pool (violaxanthin + antheraxanthin + zeaxanthin) were markedly reduced in *chaos* and *prors1-1 chaos* mutants. In contrast, β-carotene, which associates with PSII-core proteins, showed only a marginal decrease in mutant leaves, confirming the specific decrease in levels of PSII antenna proteins in the mutants containing the *chaos* allele.

**Table 2:** **Levels of leaf pigments in light-adapted mutant (*prors1-1, chaos*, and *prors1-1 chaos*) and WT (Col-0) plants at the 6-leaf rosette stage**.

	**Leaf pigment content (pmol/mg leaf)**							
	**Nx**	**Lut**	**Chl b**	**Chl a**	**β-Car**	**VAZ**	**Chl a + b**	**Chl a/b**
Col-0	60 ± 5	197 ± 15	580 ± 34	1574 ± 54	211 ± 7	66 ± 5	2154 ± 53	2.71 ± 0.09
*prors1-1*	45 ± 3	154 ± 10	480 ± 28	1225 ± 71	121 ± 8	66 ± 1	1705 ± 68	2.55 ± 0.15
*chaos*	29 ± 1	103 ± 9	197 ± 6	728 ± 34	176 ± 7	34 ± 3	925 ± 40	3.69 ± 0.08
*prors1-1 chaos*	24 ± 3	92 ± 9	165 ± 12	497 ± 39	174 ± 10	43 ± 4	742 ± 61	3.01 ± 0.09

Leaf pigments were quantified by HPLC and are reported in pmol/mg leaf (fresh weight). Mean values ± SD are shown. Nx, neoxanthin; Lut, lutein; Chl b, chlorophyll b; Chl a, chlorophyll a; β-Car, β-carotene; VAZ, violaxanthin + antheraxanthin + zeaxanthin.

Taken together, the data indicate that, in the *prors1-1 chaos* double mutant, the reduction in the size of the PSII antenna caused by the *chaos* mutation, and the resulting decrease in the total amount of light absorbed, effectively counteracts the increase in thylakoid electron pressure due to *prors1-1*. Hence, the excessive reduction of the thylakoid electron transport chain revealed by the increased 1-qL values in the *prors1-1* mutant is alleviated in *prors1-1 chaos* plants.

### Levels of light-harvesting complexes and thylakoid phosphorylation are reduced in *prors1-1 chaos* leaves

**Figure 3:**
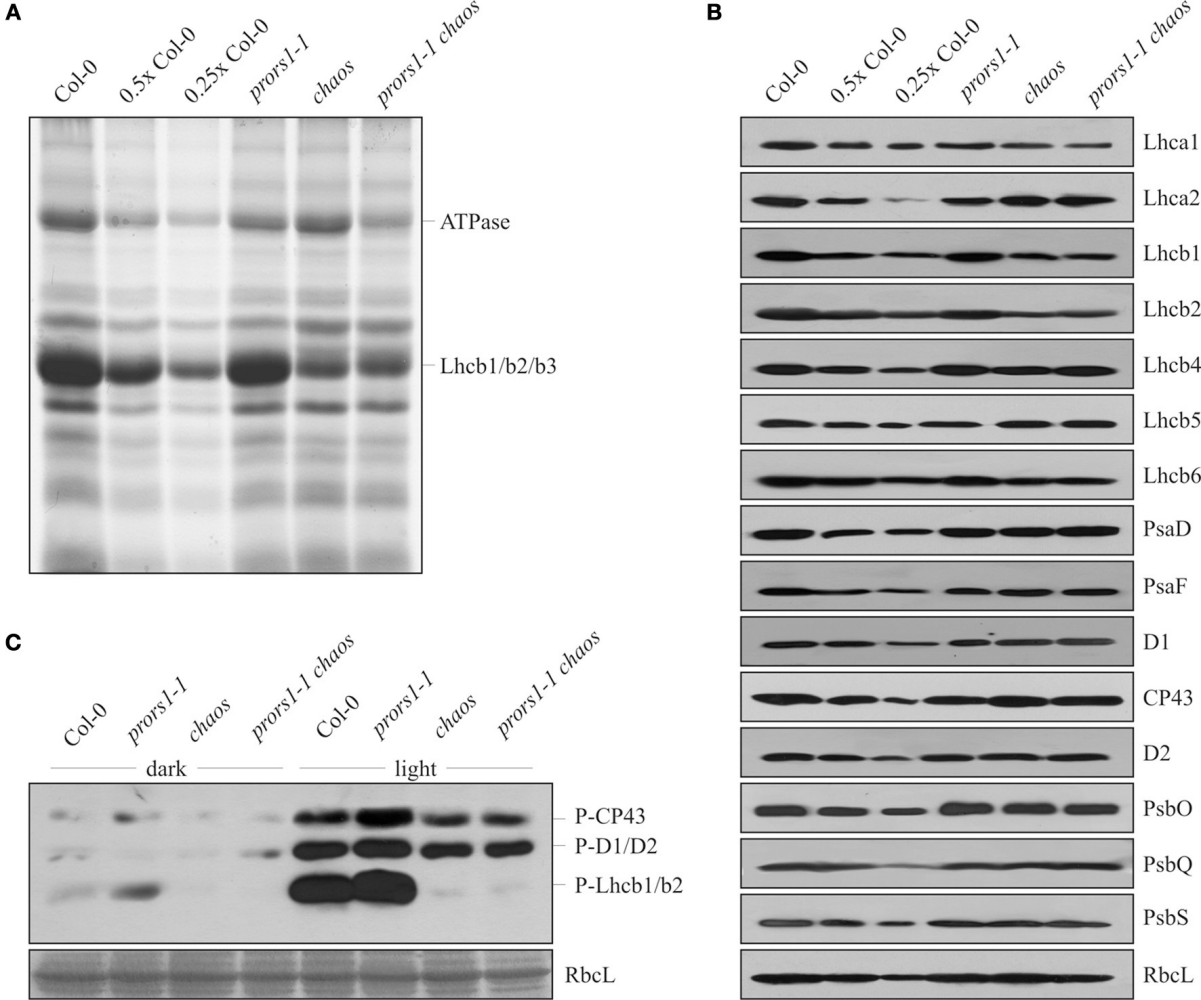
**1D SDS-PAGE and immunoblot analysis of thylakoid proteins from WT (Col-0) and mutant (*prors1-1*, *chaos*, and *prors1-1 chaos*) leaves. (A)** Thylakoid proteins isolated from equal amounts of leaf (fresh weight) were fractionated by SDS-PAGE and stained with Coomassie Brilliant blue. Band intensities were quantified by densitometric analyses (see “Materials and Methods”). **(B)** Identical amounts of total leaf proteins were fractionated by SDS-PAGEs as in **(A)**, transferred onto nylon filters and probed with antibodies raised against individual subunits of PSI (PsaD, PsaF), PSII (D1, CP43, D2, PsbO, PsbQ, PsbS), LHCI (Lhca1, Lhca2) and LHCII (Lhcb1, Lhcb2, Lhcb4, Lhcb5, Lhcb6), and the large subunit of RubisCO (RbcL). The lanes marked 0.5× Col-0 and 0.25× Col-0 were loaded with the indicated dilutions of the standard control sample. **(C)** Identical amounts of total proteins isolated from dark- and light-adapted plants were fractionated by SDS-PAGE as in **(A)**, blotted onto nylon filters and probed with a phosphothreonine (P-Thr)-specific antibody. A portion of the Coomassie Brilliant Blue-stained gel, corresponding to the RbcL migration region, was used to check for equal loading.

To determine the effects of the combined action of the *chaos* and *prors1-1* mutations on thylakoid protein composition, membranes isolated from WT and mutant plants were fractionated by 1D SDS-PAGE (Figure 3A). As expected, densitometric analyses revealed a decrease of about 70% for the major light-harvesting complex of PSII (Lhcb1, Lhcb2, Lhcb3) in both *chaos* and *prors1-1 chaos* plants (Table 3). In addition, *prors1-1* and *prors1-1 chaos* thylakoids were characterized by a decrease in the α- and β-subunits of the ATPase complex by 47 and 56%, respectively. A more detailed picture of thylakoid protein composition in WT and mutant plants was obtained through immunoblot analysis (Figure 3B, Table 3). In particular, some PSII antenna proteins, such as Lhcb1, Lhcb2, and Lhcb6, showed similar reductions in both *chaos* and *prors1-1 chaos* thylakoids, whereas Lhca1 accumulation in *prors1-1 chaos* appeared to be the result of an additive effect of the two single mutations. Levels of most of the other subunits analyzed, including polypeptides from the PSI antenna (Lhca2), the PSII antenna (Lhcb5), the cores of PSI (PsaD and PsaF) and PSII (D1, CP43, and D2), and the oxygen-evolving complex (PsbQ), were higher (i.e., closer to WT-like levels) in *prors1-1 chaos* than in *prors1-1* thylakoids. No marked differences were observed between WT and mutant plants with respect to the accumulation of PsbO (a subunit of the oxygen-evolving complex), PsbS (subunit of PSII involved in non-photochemical quenching of Chl fluorescence) or the large subunit of RubisCO (RbcL).

**Table 3:** **Quantification of thylakoid (phospho)proteins in light-adapted mutant (*prors1-1*, *chaos*, and *prors1-1 chaos*) and WT (Col-0) plants**.

**Protein**	***prors1-1***		***chaos***		***prors1-1 chaos***	
	**1D-PAGE**	**Immunoblot**	**1D-PAGE**	**Immunoblot**	**1D-PAGE**	**Immunoblot**
PsaD	nd	0.89 ± 0.07	nd	0.87 ± 0.08	nd	1.22 ± 0.06
PsaF	nd	0.78 ± 0.03	nd	1.12 ± 0.06	nd	1.25 ± 0.08
D1^*^	nd	0.61 ± 0.05	nd	0.96 ± 0.06	nd	1.23 ± 0.05
P-D1/D2	nd	1.67 ± 0.03	nd	0.52 ± 0.04	nd	0.64 ± 0.03
CP43^*^	nd	0.68 ± 0.05	nd	0.87 ± 0.06	nd	0.94 ± 0.08
P-CP43	nd	2.31 ± 0.06	nd	0.48 ± 0.03	nd	0.46 ± 0.03
D2^*^	nd	0.75 ± 0.07	nd	0.92 ± 0.06	nd	0.98 ± 0.07
PsbO	nd	1.12 ± 0.05	nd	0.97 ± 0.04	nd	1.09 ± 0.07
PsbQ	nd	0.77 ± 0.08	nd	0.91 ± 0.05	nd	1.12 ± 0.04
PsbS	nd	0.94 ± 0.05	nd	0.86 ± 0.07	nd	0.85 ± 0.08
Lhca1	nd	0.89 ± 0.05	nd	0.49 ± 0.05	nd	0.23 ± 0.06
Lhca2	nd	0.78 ± 0.03	nd	0.95 ± 0.03	nd	1.06 ± 0.08
Lhcb1/b2/b3	0.84 ± 0.05	*b*1 = 0.85 ± 0.06; *b*2 = 0.86 ± 0.04	0.31 ± 0.04	*b*1 = 0.23 ± 0.05; *b*2 = 0.32 ± 0.04	0.29 ± 0.03	*b*1 = 0.28 ± 0.04; *b*2 = 0.26 ± 0.07
P-Lhcb1/b2	nd	1.41 ± 0.09	nd	ns	nd	ns
Lhcb4	nd	1.12 ± 0.09	nd	0.79 ± 0.04	nd	0.94 ± 0.03
Lhcb5	nd	0.79 ± 0.04	nd	0.98 ± 0.06	nd	0.98 ± 0.06
Lhcb6	nd	0.81 ± 0.03	nd	0.24 ± 0.05	nd	0.33 ± 0.05
ATPase (α,β^*^)	0.63 ± 0.03	nd	0.86 ± 0.05	nd	0.44 ± 0.05	nd
RbcL^*^	nd	0.91 ± 0.04	nd	0.98 ± 0.02	nd	0.85 ± 0.06

WT levels are set to 100%. Average values were calculated from three independent 1D-PA gels and protein gel blots (see Figure 3). nd, not determined; ns, not significant.

* Proteins encoded by plastid genes. P-, phosphorylated protein. Note that levels of thylakoid protein phosphorylation in the dark are not reported, since signals were barely detectable.

The major decrease in PSII antenna proteins and the lower 1-qL values observed in *chaos* and *prors1-1 chaos* leaves imply that the thylakoid electron transport chain is more oxidized in these genotypes than in *prors1-1* plants. Because the level of thylakoid protein phosphorylation is directly linked to the reduction state of the PQ pool, immunoblot analysis using a phosphothreonine-specific antibody was carried out to investigate this further (Figure 3C). PSII-core proteins and LHCII phosphorylation were markedly increased in light-adapted *prors1-1* thylakoids, in particular when the reduced accumulation of these proteins in *prors1-1* plants (Table 3) is accounted for. On the other hand, a marked decrease of D1, D2, and CP43 phosphorylation was observed in light-adapted thylakoids isolated from *chaos* and *prors1-1 chaos* leaves. In the dark, only marginal levels of thylakoid protein phosphorylation were detected in the different genetic backgrounds.

Thus, the polypeptide composition of *prors1-1 chaos* thylakoids seems to be influenced by two major factors. Additive effects of the two single mutations *prors1-1* and *chaos* seem to act on the abundance of Lhca1, Lhcb2, and the ATPase complex, while another group of photosynthetic proteins, the abundance of which is unaffected in the *chaos* mutant but decreased in *prors1* 1(e.g., D1, PsaF, PsbQ, Lhca2, Lhcb4, and Lhcb5), behaves in a WT-like manner in *prors1-1 chaos* plants. These findings indicate (1) that the increased excitation pressure in *prors1-1* thylakoids (revealed by the increase in the 1-qL value and in thylakoid phosphorylation) markedly affects the accumulation of certain photosynthetic proteins, and (2) that this effect can be attenuated by reducing PSII antenna size through the *chaos* mutation.

### Plastid protein synthesis is unaffected by the *chaos* mutation

**Figure 4:**
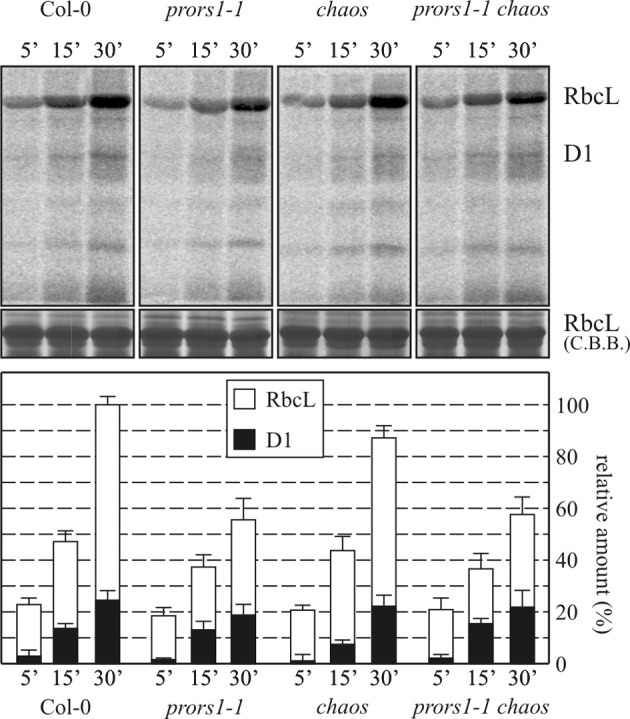
**Translational efficiency of chloroplast-encoded mRNAs in WT (Col-0) and mutant (*prors1-1*, *chaos*, and *prors1-1 chaos*) leaves.** Leaves isolated from 6-leaf-rosette plants were pulse-labeled with [^35^S]methionine under low-level illumination (20 μmol photons m^−2^ s^−1^) for 5, 15, and 30 min in the presence of cycloheximide to inhibit cytosolic protein synthesis. Total leaf proteins were then isolated, fractionated by SDS-PAGE, and detected by autoradiography. A portion of the Coomassie Brilliant Blue (CBB)-stained gel, corresponding to the RbcL migration region, was employed as internal standard for data normalization. Levels of [^35^S]methionine incorporation into RbcL and D1 proteins were quantified and are shown in the histogram. Values are averages of 5 independent experiments and were normalized to the maximal signal intensities obtained in WT leaves after 30 min labeling.

To determine whether the differences in thylakoid protein accumulation observed between *prors1-1* and *prors1-1 chaos* plants were due to *chaos*-dependent adaptive mechanisms that can modulate the translation process, plastid protein synthesis was investigated. To this end, the rate of incorporation of [^35^S]methionine into plastid proteins in young leaves of WT and mutant (*prors1-1*, *chaos* and *prors1-1 chaos*) plants was monitored for 5, 15, and 30 min in the presence of light and inhibitors of cytoplasmic protein synthesis (Figure 4). Subsequently, total leaf proteins were extracted and fractionated by SDS-PAGE. In five independent experiments, the amounts of RbcL labeled in *prors1-1* and *prors1-1 chaos* plants were comparable, and equivalent to about 55% of WT levels. A marginal decrease in PSII-D1 signals was also observed in these two mutants, whereas *chaos* plants behaved essentially like WT in this respect.

Taken together, these results exclude the possibility that the differences in thylakoid protein accumulation observed in *prors1-1* and *prors1-1 chaos* mutants result from differences in the rate of plastid protein synthesis. Therefore, the increased accumulation of plastid-encoded subunits in *prors1-1 chaos* (relative to *prors1-1*) leaves must be attributed to differences in post-translational events (including greater protein stability) associated with the lower level of oxidative damage incurred when light absorption and photosynthetic electron transport are less efficient. The nearly WT abundance of nucleus-encoded proteins observed in *prors1-1 chaos* thylakoids might be due to increases in transcription, translation, or protein stability, or any combination of these.

### The *chaos* mutation partially suppresses the OGE-dependent retrograde signaling pathway

**Figure 5:**
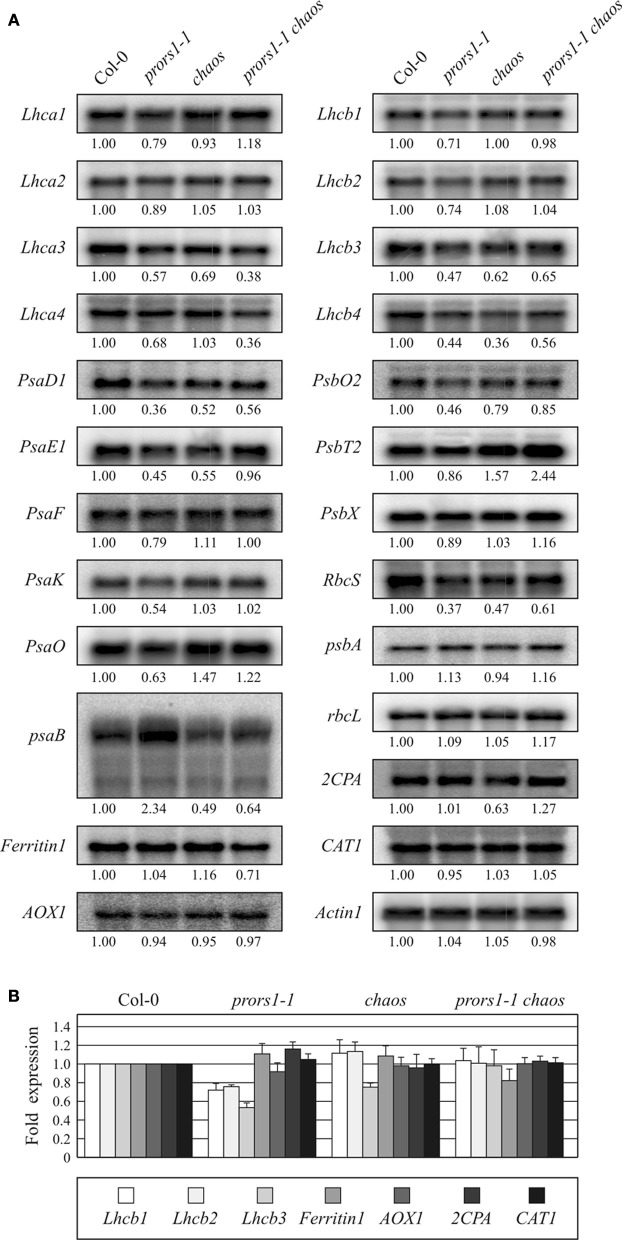
**Expression analysis of photosynthesis and antioxidant genes in WT (Col-0) and mutant (*prors1-1*, *chaos*, and *prors1-1 chaos*) leaves. (A)** Twenty microgram samples of total RNA from light-adapted WT, *prors1-1*, *chaos*, and *prors1-1 chaos* mutant plants were size-fractionated by agarose gel electrophoresis, transferred to nitrocellulose filters and probed with cDNA fragments specific for transcripts encoding subunits of PSI (*Lhca1*, *-2*, *-3*, *-4*, *psaB*, *PsaD1*, *PsaE1*, *PsaF*, *PsaK*, *PsaO*), PSII (*Lhcb1*, *-2*, *-3*, *-4*, *psbA*, *PsbO2*, *PsbT2*, *PsbX*) and Rubisco (*RbcS*, *RbcL*). Expression of nuclear genes encoding antioxidant enzymes, such as *Ferritin1*, *AOX1*, *2CPA*, CAT1, and *AOX1*, was also monitored. Three independent RNA gel-blot analyses were performed for each gene. Equal loading of RNA was checked by hybridization with *Actin1* specific probe. **(B)** Expression of genes belonging to multi-gene families, such as *Lhcb1*, *Lhcb2*, *Lhcb3*, *Ferritin1*, *AOX1*, *2CPA*, and *CAT1*, was also monitored by qRT-PCR as described in “Materials and Methods.” Average values from three biological and three technical replicates are reported. Note that the expression level of each of the gene analyzed was normalized to 1 in Col-0 background. Bars indicate standard deviations.

To assess the relative contribution of photosynthetic electron transport and the associated changes in TRS to the OGE-dependent signaling pathway, expression analyses of plastid and nuclear photosynthesis genes were conducted on WT and mutant plants. Probes for one plastid and nine nuclear genes encoding subunits of PSI and its antenna, one plastid and seven nuclear genes encoding subunits of PSII and its antenna, and for the plastid-encoded large subunit (RbcL) and the nucleus-encoded small subunit (RbcS) of RubisCO were hybridized to RNA gel blots loaded with total RNA from light-adapted leaves (Figure 5A). As expected, all nuclear photosynthesis genes were down-regulated in *prors1-1* plants, confirming the role of mitochondrial and plastid translation rate in triggering photosynthesis-related NGE (Figure 5A). However, in the *prors1-1 chaos* double mutant, 15 of the 17 nuclear photosynthesis genes analyzed were up-regulated relative to *prors1-1*. In particular, expression of *Lhca1*, *Lhca2*, *PsaE1*, *PsaF*, *PsaK*, *PsaO*, *Lhcb1*, *Lhcb2*, and *PsbX* in *prors1-1 chaos* leaves was identical to (and in the case of *PsbT2* even higher than) that in WT. The remaining genes (*PsaD1*, *Lhcb3*, *Lhcb4*, *PsbO2*, and *RbcS*) were derepressed in *prors1-1 chaos*, in some cases to levels similar to those seen in the *chaos* single mutant, but lower than in WT. Exceptions were represented by *Lhca3* and *Lhca4* genes, which were down-regulated in both *prors1-1* and *prors1-1 chaos* mutants. The limited capacity for light absorption caused by the *chaos* mutation also influences plastid gene expression, as shown by the marked drop in *psaA-B* expression in *chaos* and *prors1-1 chaos* mutants, whereas *psbA* and *RbcL* levels were almost unchanged in mutant plants.

Expression of genes involved in scavenging or preventing the formation of ROS was also investigated. The levels of transcripts of *Ferritin1*, mitochondrial alternative oxidase (*AOX1*), catalase (*CAT1*) and 2-Cys-peroxiredoxin-A (*2CPA*), expression of which has been reported to be stimulated by increases in ROS production, were only slightly altered in the mutant genotypes.

In order to validate the gene expression data obtained by Northern blot hybridizations, the expression of *Lhcb1*, *Lhcb2*, *Lhcb3*, *Ferritin1*, *AOX1*, *2CPA*, and *CAT1*, all of them belonging to large gene families, was also monitored by qRT-PCR analyses (Figure 5B). In this case too, *Lhcb* expression was specifically down-regulated in *prors1-1* leaves and derepressed in *prors1-1 chaos* plants. On the contrary, the expression of all the other genes remained unchanged between Col-0 and mutant leaves.

Taken together, the data clearly demonstrate that the OGE-dependent signaling pathway is tightly linked to photosynthetic electron transport and the associated TRS. In the *prors1-1 chaos* double mutant, the significant reduction in light absorption and oxygen evolution caused by the *chaos* mutation largely prevents the specific down-regulation of nuclear photosynthetic genes caused by the *prors1-1* mutation. Interestingly, this effect is likely to be caused by the altered redox state of the thylakoid electron transport chain (as shown by reduced levels of thylakoid protein phosphorylation and *psaA-B* operon expression), not by a burst of ROS production, as indicated by the limited changes in the expression of ROS-induced genes.

## Discussion

Attempts to distinguish between different retrograde signaling pathways have been hampered by difficulties in discriminating between primary and secondary effects caused by chemical inhibitors, and a comparative lack of genetic mutants that influence organellar function in a specific, well-defined manner (Leister, 2012; Barajas-Lopez et al., in press). In this study we have investigated the retrograde signaling pathway(s) active in *prors1-1* mutant plants, which exhibit perturbations in two chloroplast functions relevant for retrograde signaling: (1) a mild defect in protein synthesis in both mitochondria and chloroplasts; and (2) altered photosynthetic electron transport (this manuscript; Pesaresi et al., 2006).

### Diminished light absorption mitigates the defect in photosynthetic electron transport in *prors1-1* plants

The excessively reduced state of the thylakoid electron transport chain observed in the *prors1-1* mutant, as indicated by increased thylakoid protein phosphorylation, reduced effective quantum yield of PSII, and increased 1-qL values, supports the notion that many alterations in plant cell metabolism directly or indirectly impinge on the redox state of photosynthetic electron transport components, making the photosynthetic apparatus a major sensor of physiological imbalances (Pfannschmidt and Yang, 2012). Therefore, changes in thylakoid excitation pressure may be associated with major modifications in gene expression at the organellar and nuclear levels. Here, we have specifically addressed this issue by introducing the *chaos* mutation into *prors1-1* plants. This mutation in the *CAO* gene reduces the size of the PSII antenna, thus mimicking a major adaptive mechanism that plants have evolved to protect themselves against the damaging effects of excess light energy (for a review see: Oelze et al., 2008). Down-regulation of the *CAO* gene, which codes for the cpSRP43 subunit involved in the insertion of Lhcb proteins into the thylakoids (Klimyuk et al., 1999), has actually been implicated in the system that remodels the photosynthetic machinery to safeguard against photo-oxidative stress (Klenell et al., 2005). And indeed, in *prors1-1 chaos* double mutant plants, the thylakoid electron transport chain is more highly oxidized, as indicated by the higher Φ_II_ and lower 1-qL values, as well as reduced O_2_ production relative to *prors1-1* plants, and the concomitant reduction in thylakoid protein phosphorylation. These data support previous findings where *chaos* seedlings have been reported to be highly tolerant to photooxidative stress under both tightly controlled laboratory conditions and highly variable conditions in the field, and to mitigate the effects of the *lesion simulating disease 1* (*lsd1*) mutation (Mateo et al., 2004; Klenell et al., 2005). These effects are attributable to a decrease in light absorption resulting from the limited availability of proteins of the major light-harvesting complex of PSII caused by the *chaos* mutation. Immunoblot and pigment content analyses confirm that steady-state levels of Lhcb1, Lhcb2, Lhcb3, and the minor antenna Lhcb6 are markedly reduced in *chaos* and *prors1-1 chaos* mutants (this manuscript; Klimyuk et al., 1999). The observation that transcripts of the plastid *psaA-B* operon are less abundant in *chaos* and *prors1-1 chaos* leaves, but higher in *prors1-1* chloroplasts, than in WT supports the idea that the PQ pool is more oxidized in the presence of the *chaos* mutation, because expression of this operon is promoted by a reduced PQ pool (Pfannschmidt et al., 1999a,b; Allen and Pfannschmidt, 2000). Despite the partial reversal of the hyper-reduction of the thylakoid electron transport chain, the *prors1-1 chaos* plants showed a reduction of about 50% in growth rate relative to *prors1-1* single mutants under growth chamber conditions, most probably as a result of the constraints on O_2_ evolution, and consequently on ATP and NADPH production.

### Light absorption and the influence of TRS on the OGE-dependent retrograde pathway

The alleviation of the hyper-reduction of the thylakoid electron transport chain caused by the *prors1-1* mutation, together with the persistence of the defect in protein synthesis in plastids and mitochondria, as demonstrated by *in vivo* labeling assays (see Figure 4), make *prors1-1 chaos* plants ideal material for distinguishing between OGE- and TRS-dependent retrograde signals. Analyses of nuclear genes which are down-regulated in *prors1-1* (this manuscript; Pesaresi et al., 2006), and form part of a dynamic inter-compartmental transcriptional network dedicated to adjusting the activity of organelles in response to the cellular energy state and environmental stress (Biehl et al., 2005; Leister et al., 2011), support the notion that TRS and OGE play important roles in retrograde signaling. The *prors1-1 chaos* plants show a general derepression of nuclear photosynthetic genes relative to the *prors1-1* mutant. More specifically, the genes affected could be divided into four major groups: (1) genes transcribed at higher levels than in WT (like *PsbT2*), (2) genes transcribed at WT levels (*Lhca1*, *Lhca2*, *PsaE1*, *PsaF*, *PsaK*, *PsaO*, *Lhcb1*, *Lhcb2*, and *PsbX*), (3) genes expressed at levels higher than in *prors1-1* but lower than in WT leaves (*PsaD1*, *Lhcb3*, *Lhcb4*, *PsbO2*, and *RbcS*), and (4) genes whose expression was not derepressed by the *chaos* mutation (*Lhca3* and *Lhca4*). Hence, in the *prors1-1 chaos* double mutant, the decrease in the TRS stimulus (caused by the *chaos* mutation) neutralizes some of the gene regulatory effects of the *prors1-1* mutation. This observation allows us to conclude that these genes are regulated by TRS (and possibly also indirectly by the chloroplast redox state and the associated carbon metabolism), or synergistically by TRS and OGE. Indeed, we would argue that TRS and OGE signals together contribute to retrograde signaling, although the degree to which their action is synergistic varies from gene to gene. Interestingly, the transcriptional derepression effect observed in *prors1-1 chaos* leaves is associated with increased accumulation of most of the corresponding gene products, indicating the physiological significance of this regulation of transcript abundance. Thus, with the major exception of proteins whose accumulation requires their cpSRP43-mediated, post-translational insertion into thylakoids (i.e., Lhcb subunits), the levels of other photosynthetic proteins analyzed were generally higher in the *prors1-1 chaos* double mutant than in *prors1-1*, and essentially identical to those seen in WT (see Figure 2 and Table 3).

However, it should not be forgotten that the down-regulation of nuclear photosynthesis genes in *prors1-1* leaves persists even after dark adaptation, which would suggest that the OGE-dependent retrograde pathway is largely independent of light, and thus of photosynthesis and TRS (Pesaresi et al., 2006). However, this is more likely to be an example of “systemic acquired acclimation” (SAA), where plants retain a “memory” of stress conditions induced by environmental or, as in the case of *prors1-1*, genetic factors, which allows them to mount a more effective defence against further episodes of such a stress (Karpinski et al., in press). The phenomenon is triggered by systemic redox changes in the thylakoid electron transport chain, and it avoids the need for *prors1-1* plants to induce the response to excess excitation energy *de novo* every time a dark-to-light transition takes place, thus saving energy for other metabolic pathways.

Because changes in OGE inevitably affect TRS and may also result in ROS-mediated oxidative stress, TRS and/or ROS might contribute directly to OGE signaling. However, the fact that there is no change in the expression of genes involved in detoxifying ROS, such as *Ferritin1*, *2CPA*, *CAT1* and *AOX1*, argues against the possibility that the OGE signaling pathway is triggered by oxidative stress in *prors1-1* plants (this manuscript; Pesaresi et al., 2006). Nonetheless, an involvement of ROS, in particular of H_2_O_2_, in OGE signaling cannot be entirely ruled out, since studies of isolated thylakoid membranes and intact chloroplasts have shown that a fraction of the plastid-produced H_2_O_2_ reaches the cytosol even under low-light conditions, which argues that physiological levels of H_2_O_2_ may play a role in signaling (Bienert et al., 2007; Mubarakshina et al., 2010).

On the other hand, changes in photosynthetic electron transport and the associated alteration of the redox state of PQ pool and stromal compounds *per se*, cannot explain the coordinated down-regulation of nuclear photosynthesis gene expression observed in *prors1-1* leaves. This is because only the simultaneous impairment of mitochondrial and plastid OGE results in the down-regulation of most of the nuclear photosynthetic genes in this genotype (Pesaresi et al., 2006). Indeed, light-shift experiments in combination with DCMU treatments have demonstrated that only 54 nuclear Arabidopsis genes are under the direct control of the PQ redox state, and only two of these codes for components directly associated with photosynthesis (Fey et al., 2005). Moreover, the derepression of photosynthesis gene expression in *prors1-1 chaos* leaves is only partial (the accumulation of *Lhca3*, *Lhca4*, *PsaD1*, *Lhcb3*, *Lhcb4*, *PsbO2*, and *RbcS* transcripts was not restored to WT levels), implying the existence of multiple signal sources in both plastids and mitochondria, which must be integrated to enable the re-orchestration of photosynthetic NGE.

Since, photosynthetic electron transport is responsible of synthesis of carbohydrates that, in turn, are consumed in mitochondria by respiration, sugars may be also regarded as signaling metabolites involved in retrograde communication (Baier and Dietz, 2005). Thus, increased levels of glucose or sucrose repress photosynthesis gene expression, by involving hexokinase that is critical for sensing and responding to hexose signals, intracellularly (Jang et al., 1997; Rolland et al., 2006). Moreover, ABA has been also proposed to play a role in retrograde signaling, due to the fact that ABA biosynthesis starts into the chloroplasts and that ABA is strictly interconnected with photosynthesis (Baier et al., 2004). As a matter of fact, recent findings have placed the transcription factor ABI4 at the crossroads between mitochondrial and chloroplast retrograde signaling pathways and perhaps as a convergence point for mitochondria-plastid-nucleus coordination (Giraud et al., 2008). Nevertheless, *prors1-1* plants appear to be able to maintain a memory of stress conditions, suggesting that also chromatin remodeling factors, and not only transcription factors, may have a prominent role in retrograde signaling.

### Conflict of interest statement

The authors declare that the research was conducted in the absence of any commercial or financial relationships that could be construed as a potential conflict of interest.
